# Association of Pulmonary Hypertension and Monoclonal Gammopathy of Undetermined Significance

**DOI:** 10.1155/2022/8918959

**Published:** 2022-11-18

**Authors:** Melissa A. Lyle, Subir Bhatia, Eric Fenstad, Darrell Schroeder, Robert B. McCully, Martha Q. Lacy, Wayne Feyereisn

**Affiliations:** ^1^Division of Advanced Heart Failure and Transplantation, Mayo Clinic, Jacksonville, FL, USA; ^2^Department of Cardiology, Harbor UCLA Medical Center, Torrance, CA, USA; ^3^Department of Cardiovascular, Medicine Ascentia Health, Brainerd, MN, USA; ^4^Department of Quantitative Health Science, Mayo Clinic, Rochester, Minnesota, USA; ^5^Department of Cardiology, Mayo Clinic, Rochester, MN, USA; ^6^Department of Hematology, Mayo Clinic, Rochester, Minnesota, USA; ^7^Department of Medicine, Mayo Clinic, Rochester, Minnesota, USA

## Abstract

**Objective:**

To determine the prevalence of monoclonal gammopathy of undetermined significance (MGUS) in patients with PH as well as precapillary PH.

**Methods:**

Olmsted County residents with PH, diagnosed between 1/1/1995 and 9/30/2017, were identified, and age and sex were matched to a normal control group. The PH group and normal control group were then cross-referenced with the Mayo Clinic MGUS database. Charts were reviewed to verify MGUS and PH. Heart catheterization data were then analyzed in these patients for reference to the gold standard for diagnosis.

**Results:**

There were 3419 patients diagnosed with PH by echocardiography between 1995 and 2017 in Olmsted County that met the criteria of our study. When the PH group (*N* = 3313) was matched to a normal control group (3313), a diagnosis of MGUS was significantly associated with PH 10.2% (OR = l.84 [95% CI 1.5–2.2], *p* < 0.001), compared with controls 5.8% based on echo diagnosis. Using heart catheterization data (484 patients), a diagnosis of MGUS was associated with PH 13.0% (OR = 3.94 [95% CI 2.28–6.82], *p* < 0.001). For pulmonary artery hypertension (*N* = 222), a diagnosis of MGUS was associated with PH at similar 12.2% (OR = 4.50 [95%CI 1.86–10.90], *p* < 0.001.

**Conclusions:**

There is a higher prevalence of MGUS in patients with PH and precapillary PH compared with normal controls. This association cannot be explained fully by other underlying diagnoses associated with PH. Assessing for this in patients with PH of unclear etiology may be reasonable in the workup of patients found to have PH.

## 1. Introduction

Pulmonary hypertension (PH) is defined by a mean pulmonary artery pressure (mPAP) > 25  mm·Hg at rest. As PH progresses, it results in right ventricular systolic dysfunction and eventually right ventricular failure. Pulmonary hypertension is classified as five groups based on the World Health Organization (WHO) classification system: pulmonary arterial hypertension (Group 1), pulmonary hypertension due to left heart disease (Group 2), pulmonary hypertension due to chronic lung disease (Group 3), chronic thromboembolic pulmonary hypertension (Group 4), and pulmonary hypertension due to unclear mechanisms (Group 5) [1–3]. Specifically, Group 5.1 includes hematologic disorders. Updates to the clinical classification of PH and the recent 6^th^ World Symposium on Pulmonary Hypertension suggested an mPAP of > 20 mm·Hg as abnormal. Chronic hemolytic anemia and myeloproliferative disorders are included under Group 5.1 [4]. There is not yet any specific mention of an association in the classification with any monoclonal gammopathy such as myeloma, but this would be presumed to be classified under Group 5.1. More recent work by the Task Force [4] looks at a further breakdown by hemodynamic data. A categorization of precapillary PH mPAP > 20, PAWP < 15, and PVR > 3 included members of clinical groups 1, 3, 4, and 5 above.

Pulmonary hypertension is associated with chronic myeloproliferative disorders such as myelofibrosis, essential thrombocythemia, and polycythemia vera [5]. Plasma cell dyscrasias also appear to be associated with PH. In fact, PH is a known pulmonary manifestation of POEMS (polyneuropathy, organomegaly, endocrinopathy, monoclonal gammopathy, and skin changes) syndrome, present in 27–48% of POEMS patients, often with resolution of elevated pulmonary pressures after treatment [6–8]. Association of PH with multiple myeloma (MM) [9–12] and amyloidosis have been reported [13].

Monoclonal gammopathy of undetermined significance (MGUS) is the premalignant precursor of multiple myeloma. MGUS progresses to multiple myeloma at a rate of approximately 1% per year [14–16]. The prevalence of MGUS increases with age, and a prior study reported a 5.3% prevalence of MGUS among Olmsted County residents 70 years or older [17].

Although there have been series describing PH associated with MM and other plasma cell dyscrasias, to our knowledge, there has been no prior evaluation of MGUS in PH. In this study, we sought to estimate the prevalence of MGUS in patients with echo confirmed diagnosis of PH, and right heart catheterization confirmed cases and assess whether this prevalence is higher than would be expected for individuals of similar age and sex without PH.

## 2. Methods

### 2.1. Study Population

#### 2.1.1. PH Cases

The study was approved by the Mayo Clinic Institutional Review Board (IRB Protocol 12004026). A computerized review of the electronic medical records was performed to identify Olmsted County residents seen at Mayo Clinic Rochester between 1/1/1995 and 9/30/2017 with one or more codes consistent with a potential diagnosis of PH (HTCDA codes: 04261110, 04261120, 04261210, 04262410, 04262430, and 04262431; ICD-9 codes: 416.0 and 416.8). The medical records of the identified patients were then manually reviewed to ascertain whether or not the patient met diagnostic criteria for PH, and if so, the date of the earliest PH diagnosis. PH was suggested if the right ventricular systolic pressure (RVSP) on transthoracic echocardiography was > 40 mm·Hg. RVSP was calculated using the modified Bernoulli equation (4v^2^ plus estimated right atrial pressure, where v = tricuspid regurgitant peak velocity by continuous wave Doppler) as previously demonstrated [18]. If the patient underwent a hemodynamic right heart catheterization procedure, then PH was defined by a mean pulmonary artery pressure (mPAP) >  25 mm·Hg at rest [1, 18, 19]. Precapillary PH was defined as an mPAP >25 mm·Hg and a PAWP of < or equal to 15 mm·Hg and a PVR of > or equal to 3. Clinical documentation was reviewed for relevant information regarding the diagnosis of PH as well as additional review of the echocardiographic and heart catheterization data (MAL, ERF). Features such as peak TR velocity, eccentricity index, basal LV/RV ratio RVOT acceleration time and midsystolic notching, early diastolic PR velocity, RA area, and IVC size were assessed in borderline cases.

#### 2.1.2. Controls

For each PH case, a computerized search of electronic medical records was used to create a pool of potential controls by identifying all Olmsted County residents of the same sex and age who had a medical visit at Mayo Clinic Rochester within 3 months of the diagnosis date of the PH case. From these pools of potential controls, a single control was chosen at random for each PH case (1:1 matching). For each PH case, the index date was defined as the date of their PH diagnosis, and for the corresponding control, the index date was defined as the date of their medical visit that occurred during the same time frame.

#### 2.1.3. Monoclonal Gammopathy of Undetermined Significance (MGUS)

In order to identify PH cases and controls who met diagnostic criteria for MGUS, a prospectively maintained Mayo Clinic database was used. We compared the hematology database with our ICD-9 obtained diagnoses and conducted chart reviews to verify accuracy. Serum protein electrophoresis was checked to be present if this test was specifically drawn for any reason. It was not done routinely on all patients. This was true in the pulmonary hypertension group as well as in the control group.

### 2.2. Statistical Analysis

The number and percentage of PH cases and controls diagnosed with MGUS are presented overall and according to decades of age. Since MGUS is asymptomatic and routine screening is not performed, patients may have MGUS for years before it is diagnosed. For this reason, several time intervals were used to identify patients with MGUS when assessing the association between PH and MGUS. For the primary analysis, patients were categorized as having MGUS if the MGUS diagnosis occurred prior to or within 30 days after the index date. Secondary analyses were performed with MGUS defined using time intervals that extended 5 and 10 years following the index date and also using any MGUS diagnosis through 12/31/2017 (the final date for which MGUS diagnosis information was available). In all cases, analyses were performed using conditional logistic regression taking into account the 1:1 matched case-control study design. Initial analyses were performed which included interaction terms to assess whether the association between MGUS and PH differed according to age or sex. Since a significant age-by-MGUS interaction effect was detected, supplemental analyses were performed separately for subgroups defined according to decades of age. In all cases, the results of the logistic regression analyses are summarized using the odds ratio (OR) and corresponding 95% confidence interval with odds ratios >1.0 indicating a positive association between MGUS and PH.

## 3. Results

A total of 5,440 Olmsted County residents seen at Mayo Clinic Rochester between 1/1/1995 and 9/30/2017 were identified as having one or more codes consistent with a potential diagnosis of PH. After a manual review of the electronic medical charts, 3419 of these patients met the echo diagnostic criteria for PH. Of these PH patients, 17 were excluded because the earliest date of PH diagnosis was prior to the start of the study period and 88 were excluded because they were less than 18 years of age at the time of diagnosis. Of the 3314 echo confirmed PH patients (cases), 3313 were age-matched and sex-matched to an Olmsted County resident who did not have PH. There was one male diagnosed with PH at the age of 106 years that an age-matched control could not be found for, so he was excluded.

Of the 3313 PH cases included in the analysis, 1377 (41.6%) were males and 1936 (58.4%) females. The mean age (±SD) on the index date was 72.4 ± 14.8 years (range 18 to 104 years). Compared to the controls, a significantly higher percentage of the PH cases had a diagnosis of MGUS diagnosis prior to or within 30 days following their index date (6.3% vs. 4.0%, OR = l.62 [95% C.I. 1.29 to 2.03], *P* < 0.001). The association between MGUS and PH was not found to differ between males and females (sex-by-MGUS interaction *P*=0.728). However, the association between MGUS and PH was found to be dependent upon age (age-by-MGUS interaction, *P*=0.047) with a stronger association found in those diagnosed with PH at a younger age (Table 1). Similar findings were observed when MGUS was defined as any MGUS diagnosis prior to the end of the study period (overall frequency: 10.2% vs. 5.8%, OR = l.84 [95% C 0.l. 1.53 to 2.22], *P* < 0.001; sex-by-MGUS interaction *P*=0.971, age-by-MGUS interaction *P*=0.002). For the primary analysis, which looked for a diagnosis of MGUS prior to 30 days following the index date, we performed a sensitivity analysis where we only included cases and controls who had undergone SPEP testing at some point prior to 30 days following their index date. For this analysis, we did find that the percentage of individuals who had undergone testing prior to 30 days following their index date was higher for PH cases compared to controls (1588/3313 = 48% for cases versus 1319/3313 = 40% for controls; *P* < 0.001). However, if the analysis is restricted to only those who were tested, we still find a significantly higher frequency of MGUS diagnosed among cases compared to controls (OR = 1.5, 95% C.I. (1.19, 1.90), *P* < 0.001).

Similar methods were used for the heart catheterization data. We cross-referenced our list with patients that had right heart catheterization (Tables 2 and 3). 484 patients were identified by this means with confirmed PH with an mPAP of >25, and of this group, 63 were found to have MGUS. The odds ratio was 3.94 (95% CI of 2.28–6.82) with a *P* < 0.001 (see Table 2). Within this subgroup, 222 patients had precapillary pulmonary hypertension of which 27 were found to have MGUS (Table 3). Subanalysis of this was carried out in a similar fashion looking at MGUS diagnosed within 30 days, 5 years, 10 years or ever diagnosis. The odds ratio in this group was 4.50 (95% CI (1.86–10.90 with *P* = 0.001). We again see a stronger association in younger age groups whether in **PH** or precapillary PH patients.

There was a subanalysis of the 36 patients with PH that did not meet the criteria for precapillary pulmonary hypertension which showed the following. There were 3 patients with myeloma, 2 with amyloidosis, and 3 with CLL. There were 13 females and 23 males. Analysis of the 27 patients with precapillary PH and a positive monoclonal study revealed that 5 had amyloidosis, 3 CLL, 1 myeloma, 1 Hodgkin's, 1 non- Hodgkin's, 2 myelodysplasia, and 1 AIDS. Also of those with a positive MGUS, there were 13 females and 14 males.

## 4. Discussion

Pulmonary hypertension is defined as mPAP > 25 mm·Hg at rest, assessed by right heart catheterization [2]. If PH is suspected from clinical presentation and/or physical examination, a screening transthoracic echocardiogram (TTE) is appropriate. The right ventricular systolic pressure (RVSP) is equal to the pulmonary artery systolic pressure in the absence of right ventricular outflow tract obstruction or pulmonary stenosis. This was estimated by utilizing the tricuspid regurgitant peak velocity Doppler signal, including an estimate of the right atrial pressure [18, 20, 21]. When RVSP is> 40 mm·Hg on the TTE, the remainder of the echocardiographic examination typically includes a detailed assessment of right ventricular size and systolic function, the presence and severity of tricuspid regurgitation, and also a comprehensive evaluation of left-sided cardiac structures. This helps identify appropriate patients for hemodynamic right heart catheterization to confirm the PH diagnosis, classify the PH group, stratify risk, and determine candidates for PH-directed therapies [22–24]. Other tests are needed to help determine the type of PH, including pulmonary function testing to evaluate for underlying lung disease, ventilation-perfusion lung scintigraphy to assess for chronic thromboembolic PH, and laboratory evaluation. An important component of the diagnostic algorithm for PH is the laboratory evaluation, which includes screening for an underlying connective tissue disorder, chronic liver disease, human immunodeficiency virus (HIV), and myeloproliferative disorders [25].

The WHO classification system for PH includes chronic hemolytic anemia and myeloproliferative disorders in Group 5.1 [2]. The assumption would be that plasma dyscrasias should also be included in this group. There is an established association between plasma dyscrasias and PH, particularly POEMS syndrome. Recently, there has been increased awareness of PH in POEMS syndrome, with varying reports of prevalence up to 48% [7, 8, 26]. It is thought that the increased release of vasoactive cytokines, including VEGF, plays a role in the development of PH that has also been described in multiple myeloma. A recent case report describes two cases of PH in smoldering MM. Right heart catheterization in both patients showed an rnPAP of > 25 mm·Hg with normal pulmonary arterial wedge pressures (PAWP) < 15  mm·Hg and elevated pulmonary vascular resistance (PVR) > 3 Woods units, indicating the presence of precapillary PH. Autologous stern cell transplantations were performed in both patients, and the pulmonary hypertension resolved after complete remission was achieved [9]. There have been other case reports as well describing a reversible form of precapillary PH associated with MM [10, 11, 27]. A recent retrospective study of 359 patients with MM found that of the 123 patients who underwent echocardiography, 39 (32%) had elevated RVSP suggesting PH [28]. No association was found between the presence of PH and specific myeloma features, but this study further expanded on the suggestion that PH is prevalent in MM.

Although the link between PH and POEMS syndrome and active multiple myeloma has been established, it is worthwhile to delve further into the association between PH and smoldering multiple myeloma and MGUS. The International Myeloma Working Group (IMWG) defines the diagnostic criteria for MGUS as less than 3 g/dL of M-protein in the serum, less than 10% monoclonal plasma cells in the bone marrow, and no CRAB criteria (elevated calcium, renal insufficiency, anemia, or lytic bone lesions) [14]. Patients with MGUS can be stratified into low-risk, intermediate-risk, and high-risk groups, based on the risk of progression to active myeloma, which in general is 1% per year [15]. Smoldering myeloma is the asymptomatic stage between MGUS and active myeloma. Smoldering myeloma is associated with an M-protein > 3 g/dL and/or > 10% plasma cells in the bone marrow [14]. Given smoldering myeloma and MGUS are not associated with end-organ damage by definition, management is typically focused on surveillance rather than active treatment.

As mentioned above, there have only been a few case reports describing PH in the setting of smoldering multiple myeloma (9), and to our knowledge, no study has investigated a possible association between MGUS and PH. Therefore, our study is the first to establish a clear association between MGUS and PH. We found that approximately, 10% (echo data) and 13% (heart catheterization data) of patients with PH had a concomitant diagnosis of MGUS, and there was a statistically significant association between PH and MGUS when the cohort was compared with a well-established age-matched and sex-matched control group without a diagnosis of PH. This study expands on the previous work regarding an association between plasma cell dyscrasias and PH, highlighting a significant higher prevalence of MGUS in patients with PH. Ratios of lgG, IgM, IgA, and light chains were similar to values noted previously [17].

The underlying mechanism of PH in this setting is still unclear, but the pathophysiology could involve a cytokine or humoral process since anecdotally PH has been reversible with myeloma treatment [29, 30]. This study raises the question of whether the presence of PH in smoldering myeloma and MGUS is a higher risk feature, associated with worse prognosis, and it might indicate a need for closer monitoring or the initiation of earlier treatment of the hematologic condition. Further studies regarding outcomes in patients with PH and plasma cell dyscrasias will be needed. Given that laboratory evaluation is needed when there is a new diagnosis of PH, it seems reasonable to routinely screen for a monoclonal gammopathy with serum protein electrophoresis (SPEP) with immunofixation in all patients in Group 1 (3%) or Group 5 (15%) where the cause of the PH is unknown. Adding this testing, just as adding the other blood testing recommended, add to the cost and may lead to additional studies. Based on the reversible nature of the PH in several case studies of smoldering myeloma and multiple myeloma, a SPEP appears warranted. An SPEP also is needed for case finding of underlying conditions including POEMS, amyloidosis, lymphoma's and leukemias. Further study assessing the type of monocolonal protein would only apply in those with a 2 gm/dL M-protein. Costs and the morbidity of bone marrow biopsy, skeletal surveys, and additional workup are the risks associated with this. Of patients with MGUS, this equates to a small percentage of the total which reaches this level, with a reasonable yield to determine underlying diagnoses.

Since there are cases of pulmonary hypertension that have reversible features when treating their monoclonal gammopathy as seen in reference 9, the hope is more cases may be present that can also benefit.

This study does have limitations, one being the retrospective nature of the study. Another is detection bias, that is, the more times a patient is seen, the more likelihood testing will occur.

We tried to exclude or reduce this by the comparison at the end of the result section, by looking at only those who had testing. An analysis of the patient who had right heart catheterization also confirmed the association found with our echo-related diagnoses. Also, the majority of the PH patients and the control group were Caucasian based on the population in Olmsted County thus limiting the ability to broadly apply the results to other ethnic groups. However, Mayo Clinic is a tertiary/quaternary referral center with broad patient population seeking second and third opinions from across the United States and internationally.

## 5. Conclusion

This population-based study found a statistically significant higher prevalence of MGUS associated with PH. Since previous case series have shown instances of reversible PH with treating smoldering myeloma and multiple myeloma,further prospective study appears warranted since patients that have MGUS have the potential to represent a reversible cause of PH especially those who have precapillary PH. Also, the case finding of amyloidosis, as well as underlying lymphomas and leukemias, is enhanced by this. We propose that SPEP with immunofixation be included in the standard laboratory evaluation of patients presenting with a new diagnosis of PH that potentially fall within Group1 (3%) and Group 5 (15%) which have an unclear cause at the time of presentation. Following a positive SPEP over time especially in patients with precapillary PH is reasonable for the case finding of those patients that may have a reversible form of PH with the treatment of their underlying hematologic condition.

## Figures and Tables

**Table 1:** Frequency of MGUS diagnosis among PH cases and controls.

End of interval^*∗*^	Controls			PH cases			Conditional logistic regression^*∗∗*^		
	N^†^	^#^	(%)	N^†^	^#^	(%)	OR	(95% C.I.)	p
Index date + 30 days									
All ages	3313	132	(4.0)	3313	208	(6.3)	1.62	(1.29, 2.03)	<0.001
≤49	274	0	(0.0)	274	10	(3.6)	∞		
50–59	321	5	(1.6)	321	12	(3.7)	2.40	(0.85, 6.81)	0.100
60–69	569	10	(1.8)	569	26	(4.6)	2.60	(1.25, 5.39)	0.010
70–79	915	46	(5.0)	915	59	(6.4)	1.33	(0.88, 2.02)	0.174
80–89	972	57	(5.9)	972	76	(7.8)	1.34	(0.95, 1.89)	0.098
≥90	262	14	(5.3)	262	25	(9.5)	1.85	(0.94, 3.63)	0.075

Index date + 5 years									
All ages	3313	164	(5.0)	3313	303	(9.1)	1.95	(1.60, 2.37)	<0.001
≤49	274	1	(0.4)	274	14	(5.1)	14.0	(1.84, 106.5)	0.011
50–59	321	6	(1.9)	321	24	(7.5)	4.00	(1.64, 9.79)	0.002
60–69	569	15	(2.6)	569	45	(7.9)	3.14	(1.72, 5.74)	<0.001
70–79	915	59	(6.4)	915	85	(9.3)	1.53	(1.07, 2.19)	0.021
80–89	972	66	(6.8)	972	106	(10.9)	1.66	(1.21, 2.28)	0.002
≥90	262	17	(6.5)	262	29	(11.1)	1.75	(0.95, 3.23)	0.074

Index date + 10 years									
All ages	3313	184	(5.6)	3313	327	(9.9)	1.87	(1.55, 2.56)	<0.001
≤49	274	1	(0.4)	274	17	(6.2)	17.0	(2.26, 127.7)	0.006
50–59	321	7	(2.2)	321	25	(7.8)	3.57	(1.55, 8.26)	0.003
60–69	569	21	(3.7)	569	51	(9.0)	2.50	(1.49, 4.20)	<0.001
70–79	915	64	(7.0)	915	97	(10.6)	1.62	(1.15, 2.29)	0.006
80–89	972	74	(7.6)	972	108	(11.1)	1.50	(1.10, 2.04)	0.010
≥90	262	17	(6.5)	262	29	(11.1)	1.75	(0.95, 3.23)	0.074

Ever diagnosed^‡^									
All ages	3313	193	(5.8)	3313	339	(10.2)	1.84	(1.53, 2.22)	<0.001
≤49	274	1	(0.4)	274	18	(6.6)	18.0	(2.40, 134.8)	0.005
50–59	321	8	(2.5)	321	28	(8.7)	3.50	(1.60, 7.68)	0.002
60–69	569	25	(4.4)	569	56	(9.8)	2.35	(1.44, 3.83)	<0.001
70–79	915	66	(7.2)	915	99	(10.8)	1.60	(1.14, 2.24)	0.006
80–89	972	76	(7.8)	972	109	(11.2)	1.47	(1.09, 1.99)	0.013
≥90	262	17	(6.5)	262	29	(11.1)	1.75	(0.95, 3.23)	0.074

^*∗*^For the primary analysis, MGUS diagnoses made prior to the index date + 30 days were included. Since patients may have MGUS for years before being diagnosed, sensitivity analyses were performed which used various extended time intervals to identify patients with MGUS. Note that since MGUS diagnoses were only available through 12/31/2017, the duration of time interval was extended beyond the index date which was truncated for some patients. ^†^ Number of PH cases and controls in the given age category. ^‡^ Any MGUS diagnosis prior to 12/31/2017. ^*∗∗*^Conditional logistic regression taking into account the 1:1 matched set study design.

**Table 2:** Frequency of MGUS diagnosis among 484 PH cases with right heart catheterization and their matched controls.

End of interval^*∗*^	Controls			PH cases			Conditional logistic regression^*∗∗*^		
	*N* ^†^	^#^	(%)	*N* ^†^	^#^	(%)	OR	(95% C.I.)	*P*
Index date + 30 days									
All ages	484	9	(1.9)	484	29	(6.0)	3.22	(1.53, 6.81)	0.002
≤49	86	0	(0.0)	86	2	(2.3)			
50–59	96	1	(1.0)	96	4	(4.2)			
60–69	122	2	(1.6)	122	4	(3.3)			
70–79	119	5	(4.2)	119	10	(8.4)			
80–89	55	1	(1.8)	55	8	(14.5)			
≥90	6	0	(0.0)	6	1	(16.7)			

Index date + 5 years									
All ages	484	11	(2.3)	484	48	(9.9)	4.36	(2.27, 8.40)	<0.001
≤49	86	0	(0.0)	86	3	(3.5)			
50–59	96	1	(1.0)	96	7	(7.3)			
60–69	122	2	(1.6)	122	8	(6.6)			
70–79	119	6	(5.0)	119	18	(15.1)			
80–89	55	2	(3.6)	55	11	(20.0)			
≥90	6	0	(0.0)	6	1	(16.7)			

Index date + 10 years									
All ages	484	14	(2.9)	484	57	(11.8)	4.01	(2.27, 7.31)	<0.001
≤49	86	0	(0.0)	86	4	(4.7)			
50–59	96	1	(1.0)	96	8	(8.3)			
60–69	122	2	(1.6)	122	11	(9.0)			
70–79	119	7	(5.9)	119	22	(18.5)			
80–89	55	4	(7.3)	55	11	(20.0)			
≥90	6	0	(0.0)	6	1	(16.7)			

Ever diagnosed^‡^									
All ages	484	16	(3.3)	484	63	(13.0)	3.94	(2.28, 6.82)	<0.001
≤49	86	0	(0.0)	86	4	(4.7)			
50–59	96	1	(1.0)	96	10	(10.4)			
60–69	122	3	(2.5)	122	15	(12.3)			
70–79	119	8	(6.7)	119	22	(18.5)			
80–89	55	4	(7.3)	55	11	(20.0)			
≥90	6	0	(0.0)	6	1	(16.7)			

^*∗*^For the primary analysis, MGUS diagnoses made prior to the index date + 30 days were included. Since screening for MGUS was not conducted routinely, sensitivity analyses were performed which used various extended time intervals when identifying patients with a diagnosis of MGUS. Since MGUS diagnoses were only available through 12/31/2017, the duration of the extended intervals was truncated for some patients. ^†^ Number of PH cases and controls in the given age category. ^‡^ Any MGUS diagnosis prior to 12/31/2017. ^*∗∗*^Conditional logistic regression taking into account the 1:1 matched set study design.

**Table 3:** Frequency of MGUS diagnosis among 222 PH cases using restrictive right heart catheterization criteria and their matched controls.

End of interval^*∗*^	Controls			PH cases			Conditional logistic regression^*∗∗*^		
	*N* ^†^	^#^	(%)	*N* ^†^	^#^	(%)	OR	(95% C.I.)	*P*
Index date + 30 days									
All ages	222	4	(1.8)	222	14	(6.3)	3.50	(1.15, 10.63)	0.027
≤49	43	0	(0.0)	43	1	(2.3)			
50–59	45	0	(0.0)	45	3	(6.7)			
60–69	57	1	(1.8)	57	2	(3.5)			
70–79	50	2	(4.0)	50	3	(6.0)			
80–89	26	1	(3.8)	26	5	(19.2)			
≥90	1	0	(0.0)	1	0	(0.0)			

Index date + 5 years									
All ages	222	4	(1.8)	222	23	(10.4)	5.75	(1.99, 16.27)	0.001
≤49	43	0	(0.0)	43	2	(4.7)			
50–59	45	0	(0.0)	45	5	(11.1)			
60–69	57	1	(1.8)	57	4	(7.0)			
70–79	50	2	(4.0)	50	5	(10.0)			
80–89	26	1	(3.8)	26	7	(26.9)			
≥90	1	0	(0.0)	1	0	(0.0)			

Index date + 10 years									
All ages	222	5	(2.3)	222	25	(11.3)	5.00	(1.91, 13.06)	0.001
≤49	43	0	(0.0)	43	2	(4.7)			
50–59	45	0	(0.0)	45	5	(11.1)			
60–69	57	1	(1.8)	57	5	(8.1)			
70–79	50	2	(4.0)	50	6	(12.0)			
80–89	26	2	(7.7)	26	7	(26.9)			
≥90	1	0	(0.0)	1	0	(0.0)			

Ever diagnosed^‡^									
All ages	222	6	(2.7)	222	27	(12.2)	4.50	(1.86, 10.90)	0.001
≤49	43	0	(0.0)	43	2	(4.7)			
50–59	45	0	(0.0)	45	5	(11.1)			
60–69	57	2	(3.5)	57	7	(12.3)			
70–79	50	2	(4.0)	50	6	(12.0)			
80–89	26	2	(7.7)	26	7	(26.9)			
≥90	1	0	(0.0)	1	0	(0.0)			

^*∗*^For the primary analysis, MGUS diagnoses made prior to the index date + 30 days were included. Since screening for MGUS was not performed routinely, sensitivity analyses were performed which used various extended time intervals when identifying patients with a diagnosis of MGUS. Since MGUS diagnoses were only available through 12/31/2017, the duration of the extended intervals was truncated for some patients. ^†^ Number of PH cases and controls in the given age category. ^‡^ Any MGUS diagnosis prior to 12/31/2017. ^*∗∗*^Conditional logistic regression taking into account the 1:1 matched set study design.

## Data Availability

The data that support the findings of this study are available on request from the corresponding author. The data are not publicly available due to privacy or ethical restrictions.

## Abbreviations

MGUS: Monoclonal gammopathy of undetermined significance
MM: Multiple myeloma
mPAP: Mean pulmonary artery pressure
PAH: Pulmonary artery hypertension
PCWP: Pulmonary capillary wedge pressure
PH: Pulmonary hypertension
PVR: Pulmonary vascular resistance
RV: Right ventricle
RVSP: Right ventricular systolic pressure
SM: Smoldering myeloma
WHO: World Health Organization.
